# Toxicological Analysis in Tissues Following Exhumation More Than Two Years after Death (948 Days): A Forensic Perspective in a Fatal Case

**DOI:** 10.3390/toxics11060485

**Published:** 2023-05-26

**Authors:** Giuseppe Davide Albano, Stefania Zerbo, Corinne La Spina, Mauro Midiri, Daniela Guadagnino, Tommaso D’Anna, Roberto Buscemi, Antonina Argo

**Affiliations:** Section of Legal Medicine, Department of Health Promotion, Mother and Child Care, Internal Medicine and Medical Specialties, University of Palermo, 90129 Palermo, Italy

**Keywords:** exhumation, proteomic analysis, forensic, postmortem interval, sudden cardiac death

## Abstract

Exhumations are performed in accordance with a court order and are crucial instruments in the investigation of death allegations. When a death is thought to be the result of drug misuse, pharmaceutical overdose, or pesticide poisoning, this process may be used on human remains. However, after a protracted postmortem interval (PMI), it might be difficult to detect the cause of death by looking at an exhumed corpse. The following case report reveals problems associated with postmortem drug concentration changes following exhumation more than two years after death. A 31-year-old man was found dead in a prison cell. Onan inspection of the place, two blister packs, one with a tablet and the other empty, were taken and kept by the police officers. The evening before, the deceased would have taken cetirizine and food supplements consisting of carnitine–creatine tablets. No relevant autopsy findings have been observed. The toxicological analysis was performed by gas chromatography coupled to mass spectrometry and was negative for substances of abuse. Proteomic analysis was positive for creatine detection and negative for other drugs (clarithromycin, fenofibrate, and cetirizine). The presented case shows the methods, the findings, and the limitations of toxicological analysis in an exhumation case with a long postmortem interval (PMI).

## 1. Introduction

An exhumation is an important tool in investigating suspicions of death, carried out under court order. It involves unburying a body, performing an autopsy, and evaluating all remains found near the body [1,2,3,4]. This procedure may be performed on human remains in the case of a death suspected to be due to poisoning with pesticides, medications, or drug abuse [3,5,6,7,8]. However, it can be challenging to diagnose by examining an exhumed corpse after a prolonged PMI. It may be difficult to differentiate actual lesions from autolytic changes, which should be carefully assessed. Moreover, postmortem toxicology investigations on exhumed human remains are rare but a true challenge for both the pathologist and the forensic toxicologist. Some drugs’ concentrations may be influenced by the activity of certain enzymes, even after death [9]. Moreover, autolysis may cause the release of exogenous compounds [10]. Bacteria metabolism may influence the toxicological analysis results by using particular drugs or metabolites as substrates [11]. Furthermore, redistribution after death is influenced by the postmortem interval, even in the post-sampling and storage periods [12,13]. Previous reports stated the issues and limitations of toxicological analysis in exhumation cases, highlighting that conventional biological matrices may not be available due to putrefaction processes and that different postmortem phenomena may contribute to the reliability of toxicological findings [14,15].Therefore, toxicological analysis of exhumed human remains is still a significant challenge. In this case report, we report our forensic investigation experience of a death in prison for which toxicological investigations were requested two years after the death. Prior to an exhumation, the question usually arises whether presumed morphological or toxicological findings can be detected after certain postmortem intervals. The presented case shows the methods, the findings, and the limitations of toxicological analysis in exhumation cases with a long postmortem interval (PMI).

## 2. Case Report

### 2.1. Case History

This is the case of a 31-year-old man found dead in a prison cell. On an inspection of the place, two blister packs, one with a tablet and the other empty, were taken and kept by the police officers. The evening before, the deceased would have taken cetirizine and food supplements consisting of carnitine–creatine tablets. A study of the documentation revealed in the anamnesis the presence of exertional precordialgiaextra-systolic arrhythmic activity, isolated ventricular premature beats (BEV), gastritis, and papulo-pustular acne.

Following this first judicial inspection, an autopsy examination was ordered, and thecompetent judicial authority (J.A.) ordered the dismissal of the case. However, more than two years later (948 days), the defenders of the deceased’s family argued that the drugs taken the night before would have influenced the death event. Therefore, it was deemed necessary to proceed with the exhumation of the body.Among the questions posed was the need to proceed with the sampling to verify the presence of the drugs resulting from the prescribed therapy.

### 2.2. Autopsy Findings

The autoptic examination showed a male subject’s corpse, apparentlyof regular build, with indefinable somatic characteristics due to the advanced putrefactive phenomenon. When the clothes were removed, the signs of the previous autoptic activity were revealed, with resection of the skullcap and cutting of the trunk; overall, the body had an appearance tending toward corification in the head–neck district, with a great representation of the putrefactive changes in the remaining part of the human residues of the exhumed body. The following organs were recognizable: stomach, intestine, aortic tract, and kidney; the state of decomposition made their removal superfluous for subsequent histopathological evaluations. The following tissues were collected and sampled for toxicological investigations: hair matrices, quadriceps muscles of the right thigh, and tissue fragments of possible renal origin.

### 2.3. Toxicological Analysis for Proteins, Drugs and Substances of Abuse

The toxicological analysis was carried out via gas chromatographic analysis with a mass detector of the extracts, acids, and bases subjected to a derivatization process. The extraction was carried out on organic residue. About 1 g of organic material was taken and finely minced with a mechanical instrument. Then, 4 mLof distilled water was added, and the whole was left to macerate for 48 h at 4 °C.

The sample was reconditioned at room temperature. It was then stirred on a rotating stirrer for 20′ and centrifuged for 10′ at 3500 rpm; the analysis was performed on the supernatants loaded on SPE cartridges bondelut certify Agilent 130 mg.The elution was performed with 2 mL of a freshly prepared mixture of dichloromethane and isopropanol containing 2% ammonia in a ratio of 78/20/2. The eluate was collected in a glass test tube containing 50 μLof a methanol/HCl mixture (9:1). The internal standard, i.e., a solution of the deuterated compounds, was added to the eluate before it dried.

The analysis was performed by gas chromatography technique coupled to mass spectrometry using an Agilent model 6890 N gas chromatograph connected to a model 5973 mass spectrometer operating at 70 eV with a source temperature of 230 °C; the instrument was operated using computers and Mass Hunter software.

An Agilent (Agilent Technologies Inc., Santa Clara, CA, USA) J&W DB-5 ms Ultra Inert capillary column with a length of 30 m, an internal diameter of 0.25 mm, and a film thickness of 0.25 μm was used for the chromatographic separation. The analysis was conducted with a programmed temperature starting from 150 °C for 1.00 min; then, the oven temperature was increased by 20 °C per minute up to 210 °C and maintained for 10 s; subsequently, the temperature was increasedfrom 20 °C by 10 °C per minute to reach the temperature of 250 °C, which was held for one minute. Finally, with a rise of 20 °C per minute, the temperature of 280 °C was reached and maintained for two minutes.The chromatographic run had a total duration of 11.2 min, and the data were acquired in sim (selected ion monitoring) mode. With the GC/MS technique, the identification of the substances present in the extracts occurred through the evaluation of the retention time of the gas chromatographic peak and its fragmentation spectrum; the latter was compared with those included in the computerized libraries with which the management software was equipped of the tool. Toxicological analyses were negative for substances of abuse and cetirizine presence. Analysis of the composition of food supplements (AMCO)for food products and supplements provides the hypothetical identity of the compounds present in the sample. Identification was made using the official database of the National Institute for Standards and Technologies (NIST) without the aid of a certified analytical standard. The test should be interpreted as a first-level screening. The detection of compounds is subject to the limits of instrumental detection.

The sample was digested using trypsin. An analysis was conducted in the auto-fragmentation acquisition mode. The data obtained were processed using SANIST Hb software with a generic “Uniprot” database. The results of this processingwere sent once the software had finished. An analysis was performed on thesample by looking for the compounds: creatine 132.13, detected; clarithromycin 748.47, not detected; and fenofibrate 361.11, not detected.

The sample was treated by enzymatic digestion with trypsin and analyzed in Auto Ms(n) acquisition mode. The data obtained were processed with the SANIST Hb software using a generic “Uniprot” database. From this processing, results were obtained with a statistical score of less than 30.

The sample was treated by enzymatic digestion with trypsin and analyzed in Auto Ms(n) acquisition mode. The data obtained were processed with the SANIST Hb software using a generic “Uniprot”database. From this processing, results were obtained with a statistical score of less than 30.

## 3. Discussion

In the specific case, the evening before the death, the patient had taken only one tablet of cetirizine and food supplements consisting of carnitine–creatine tablets. A single dose of carnitine can hardly reach a concentration to determine effects on cardiac repolarization, as in cases after prolonged administration, which can be evaluated by continuous activity on the QTC. 

Indeed, the drug is well absorbed by the gastrointestinal tract after oral administration. The drug is distributed to biological tissues, reaching maximum plasma concentrations within one hour. The half-life of the drug is around 8 h. Cetirizine crosses the blood–brain barrier with difficulty, which accounts for the drug’s minor sedative effects compared to other antihistamines. Plasma protein binding is 93%. Cetirizine is metabolized in the liver to a minimal extent and is an inactive metabolite. Elimination occurs by the renal route, with 60–70% of the drug eliminated in 24 h. A further 10% is excreted in the urine over 96 h. Reduced renal or hepatic function may result in slower elimination by prolonging its half-life. Half-life (T/2) is the time required for the amount of a drug in the body to decrease by 50% during elimination. Therefore, knowledge of the half-life of a drug is essential to allow adequate administration and avoid incurring an overdose. The half-life is a value independent of the drug’s concentration and our body’s metabolic activity. If the T/2 is short, then the decrease in concentration will be fast; conversely, the longer the half-life, the slighter the reduction in concentration. In daily clinical practice, the half-life is necessary to reach a specific effective plasma concentration value or to maintain a particular concentration of the plasma value of the drug for a long time (days or months) [16].

Therefore, it was assumed that after eight hours, the concentration of the drug was reduced by half, corresponding to 04.00–06.00 in the morning, the time of death, and still could not reach a stable concentration, which requires further administration over time. Therefore, it is difficult to assume that the single administered dose could have exerted such a pharmacological activity to alter the QTc and cause potential arrhythmia [17,18].

As for the carnitine and the creatine taken in the evening, it is noted that they are supplements (drugs) without adverse effects on the cardiovascular system, so much so that they are suggested as prophylactic protection in subjects receiving chemotherapy.In addition, when taken in high doses, it provides cardioprotection. In a review, the optimal amount was around 3g/day, with patients not obtaining better results even with higher doses of up to 6 g/day. Understood in the meaning of “drug,” it has almost no contraindications and nearly no side effects, so much so that it is used as an over-the-counter drug, while its use as a supplement is as an anti-fatigue [5,11,19]. Carnitine is an endogenous product; however, in this case presentation, it was suspected by parents (irrespective of well-known scientific evidence) of ingestion as a protein-enriched food, which could interfere withthe cardiac function of the dead man in jail. The prosecutor’s office asked for such an analysis.

The above-described analysis of the literature on the incidence and the potential lethality of the patient’s arrhythmic disturbances suggests the probable hypothesis that the patient’s death was caused by a malignant ventricular arrhythmic event. The event was probably caused by a pre-existing extra-systolic focus of possible genetic nature and is still not always identifiable, as well as being a potential genetic character not detectable in ECG analysis [20,21,22].

To date, the toxicological investigations carried out have not allowed the detection of substances referred to in the pharmacological anamnesis. This outcome must, in any case, be evaluated in the context of studies carried out on widely degraded tissue samples of muscle matrix more than three years after the death and the sampling itself, making this investigation very limited in the reliability of the laboratory data.

In every test, choosing a practical sample type is an important choice. It is crucial to choose the right procedure if you want results that are legitimate and satisfying. The selection process is most strongly influenced by how valuable each sample type is [22,23].

When the analytes and sample matrix are defined, the next step is to develop a chromatographic system providing highly efficient separation for the targeted analytes and the corresponding matrix [24].

These techniques range from the simplest and currently rarely used, thin-layer chromatography (TLC), to capillary electrophoresis (CE) techniques, gas chromatography (GC), high-performance liquid chromatography (HPLC), and, finally, the most sophisticated, ultra-high-performance liquid chromatography (UHPLC) [25,26].

Substrates in postmortem toxicology are often seriously influenced by post-mortal degradation, redistribution, matrix effects, temperature, etc. Therefore, interpretation of the results may be difficult, especially when toxicological examinations must be carried out following exhumation. Many issues with various elements of decomposition may arise during the study of postmortem material. First, redistribution of many medications and poisons is known to happen throughout autolysis and into the putrefactive stage of decomposition, where residual enzyme activity can continue to metabolize pharmaceuticals and poisons. During putrefaction, bacteria that enter the body break down the tissues. The ensuing matrix of protein and lipid breakdown products can make it challenging to identify and quantify analytes. The same bacteria that putrefy the body can also break down medications and toxins inside the body, altering concentrations and perhaps causing the full loss of some analytes. Several of these drug breakdown pathways and their byproducts remain unknown. Recent studies suggest a possible role for metabolomics and proteomics in forensics to provide information about the assumption of substances and the postmortem interval. However, strong evidence is still lacking, and future research is needed to validate these methodologies and their application in the forensic field [27,28]. In the literature, comprehensive lists have been published called “expectation catalogues” on morphological and toxicological findings in relation to the postmortem interval. For this reason and due to the huge variety of possible issues, it may be difficult or impossible for even an experienced forensic specialist to acquire the necessary knowledge on his own [6,7,8,29,30,31,32] (Box 1).

Box 1 **Take-home messages of toxicological and proteomic analysis in cases of autopsy tissues coming from exhumation with a high postmortem interval.**

**Key points**

1. A number of drugs are unstable either chemically (from pH changes), biochemically (from residual enzyme activity), or from use as substrates by putrefactive bacteria, or a combination of the above;

2. Postmortem redistribution of drugs and poisons increases with the postmortem interval, and peripheral blood samples should be analyzed in preference to those from central areas for quantitative purposes;

3. Byproducts of decomposition are putrefactive compounds, which can interfere with the analysis of drugs, particularly with less selective detectors;

4. The samples most frequently used in cases of toxicological investigations after exhumation with large PMI are: blood/plasma, hair, liver, and brain;

5. The most commonly used toxicological analysis techniques in cases of toxicological investigations after exhumation with large PMI are: thin-layer chromatography (TLC), capillary electrophoresis (CE) techniques, gas chromatography (GC), high performance liquid chromatography (HPLC), and finally, the most sophisticated, ultra-high-performance liquid chromatography (UHPLC);

6. Care should, therefore, be taken when interpreting quantitative drug and poison results in forensic toxicology, especially where there is evidence of putrefaction.

## 4. Conclusions

The toxicological investigations carried out did not allow the detection of substances referred to in the pharmacological anamnesis. The creatine detection was not relevant to the cause of death. This outcome must, in any case, be evaluated in the context of investigations carried out on tissue samples (largely degraded tissue fragments, mostly of the muscle matrix) taken more than two years after death, making this investigation very limited in the reliability of the laboratory data. Despite the great potential shown by these techniques, their active implementation in forensic investigations requires thorough validation studies to provide a uniform and certain interpretation of the results and strong reliability when the data are presented in a courtroom.

## Data Availability

All data are included in the main text.
